# KIAA1522 is a novel prognostic biomarker in patients with non-small cell lung cancer

**DOI:** 10.1038/srep24786

**Published:** 2016-04-21

**Authors:** Yi-Zhen Liu, Hai Yang, Jian Cao, Yan-Yi Jiang, Jia-Jie Hao, Xin Xu, Yan Cai, Ming-Rong Wang

**Affiliations:** 1 Department of Medical Oncology, Fudan University Shanghai Cancer Center, Department of Oncology, Shanghai Medical College, Fudan University, Shanghai 200032, China; 2 State Key Laboratory of Molecular Oncology, Cancer Institute/Hospital, Peking Union Medical College and Chinese Academy of Medical Sciences, Beijing, China; 3 Department of Pathology, Cancer Institute/Hospital, Peking Union Medical College and Chinese Academy of Medical Sciences, Beijing, China

## Abstract

Nowadays, no robust biomarkers have been applied to clinical practice to provide prognostic evaluation of non-small cell lung cancer (NSCLC). This study aims to identify new potential prognostic biomarkers for NSCLC. In the present work, KIAA1522 is screened out from two independent GEO datasets as aberrantly up-regulated gene in NSCLC tissues. We evaluate KIAA1522 expression immunohistochemically in 583 NSCLC tissue samples and paired non-tumor tissues. KIAA1522 displays stronger staining in NSCLC cases than in adjacent normal lung tissues. Importantly, patients with KIAA1522 overexpression had a significantly shorter overall survival compared to those with low expression (*P* < 0.00001). Multivariate Cox regression analyses show that KIAA1522 is an independent prognostic indicator, even for early-stage NSCLCs (*P* = 0.00025, HR = 2.317, 95%CI: 1.477–3.635). We also found that high expression of KIAA1522 is a significant risk factor for decreased overall survival of the patients who received platinum-based chemotherapy. Gene set enrichment analysis (GSEA) and functional studies reveal that KIAA1522 is associated with oncogenic KRAS pathways. Taken together, high expression of KIAA1522 can be used as an independent biomarker for predication of poor survival and platinum-resistance of NSCLC patients, and aberrant KIAA1522 might be a new target for the therapy of the disease.

Lung cancer is the most common cause of cancer-related death worldwide. Cancer Statistics 2015 reported that more than 221,200 new cases of lung cancer are detected and approximately 158,040 people die of the disease in the United States, which represent 13.3% of all new cancer cases and 26.8% of cancer deaths in this population^1^. In China, mortality from lung cancer has increased by 465% during the past 30 years^2^. Squamous cell carcinoma (SCC) and adenocarcinoma (ADC) of the lung are the most common subtypes. Nearly 70% of patients will be diagnosed with advanced disease that is not amenable to curative therapy^3^. Counting on all stages, only about 17% patients could survive beyond 5 years.

Increasing evidences suggest a significant role for biomarkers in evaluation of patient prognosis with NSCLC^4^,^5^,^6^. At present, most discoveries of biomarkers for NSCLCs are focused on the individual gene with known functions or a panel of genes within specific functional groups^7^,^8^,^9^,^10^,^11^, while few genes with uncharacterized biological or molecular functions are studied for their prognostic value, leaving this type of genes out of the usage as biomarkers. To evaluate the feasibility of uncharacterized genes/proteins as biomarkers for NSCLC and to identify novel prognostic and predictive biomarkers from this subset of genes, we explored a screening approach to search uncharacterized genes which are aberrantly expressed in NSCLC tissues and may play potential prognostic roles in patients with NSCLC. KIAA1522 is an uncharacterized gene screened out by this approach. However, the clinical significance of the protein remains to be unveiled.

There have been little reports about the functions of KIAA1522, besides several high-throughput screening methods hit this gene without further investigation^12^,^13^,^14^. In our previous study, we have shown that a six-protein panel containing KIAA1522 could act as diagnostic marker in the bronchial brushing specimens of the patients with lung cancer^15^. The examination of KIAA1522 following screened out by our approach in the tumor tissue samples may extend the range of utility of this protein as a biomarker in lung cancer patients.

Here, we show that the gene of KIAA1522 is aberrantly high expression in the NSCLC tissues and functions as a prognostic biomarkers indicating poor survival of NSCLC patients.

## Results

### Up-regulation of KIAA1522 expression in NSCLC

**Figure 1:**
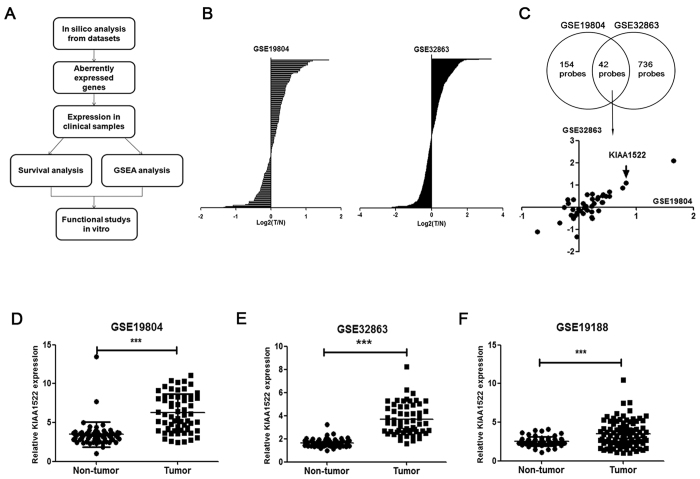
Identification of KIAA1522 as an overexpressed gene in NSCLC. (**A**) The flow chart of the present work. (**B**) Screen of uncharacterized genes (genes without GO function annotation) in GSE19804 and GSE32863 datasets. For each selected probe, the average value of log_2_ (Tumor/Non-tumor) was calculated and shown in the rank order. (**C**) The average values of log_2_ (Tumor/Non-tumor) of the genes presented in both GSE19804 and GSE32863 datasets were shown. (**D**–**F**) Transcript levels of KIAA1522 in non-tumor tissues and NSCLC tissues from GSE19804 (**D**), GSE32863 (**E**) and GSE19188 (**F**) datasets. Data are represented as scatter dot plot with mean ± s.d. (by t-test analysis, ***P < 0.001).

We used the approach shown in Fig. 1A to find novel biomarkers of lung cancer. First of all, we screened the uncharacterized genes (genes without GO annotations) that aberrantly expressed in two datasets (GSE19804 and GSE32863) of NSCLC (Fig. 1B). Among the hits of genes overlapped in the screening of the two datasets, KIAA1522 was identified to be up-regulated with positive and relatively high log_2_ (Tumor/Non-tumor) value in both datasets (Fig. 1C). Statistical analysis was also performed to confirm the overexpression of KIAA1522 in the two datasets (P < 0.0001) (Fig. 1D,E). Moreover, another NSCLC dataset (GSE19188) was also explored to verify the enhancement of KIAA1522 expression in tumor samples (Fig. 1F).

### KIAA1522 protein expression is elevated in NSCLC patients

**Figure 2:**
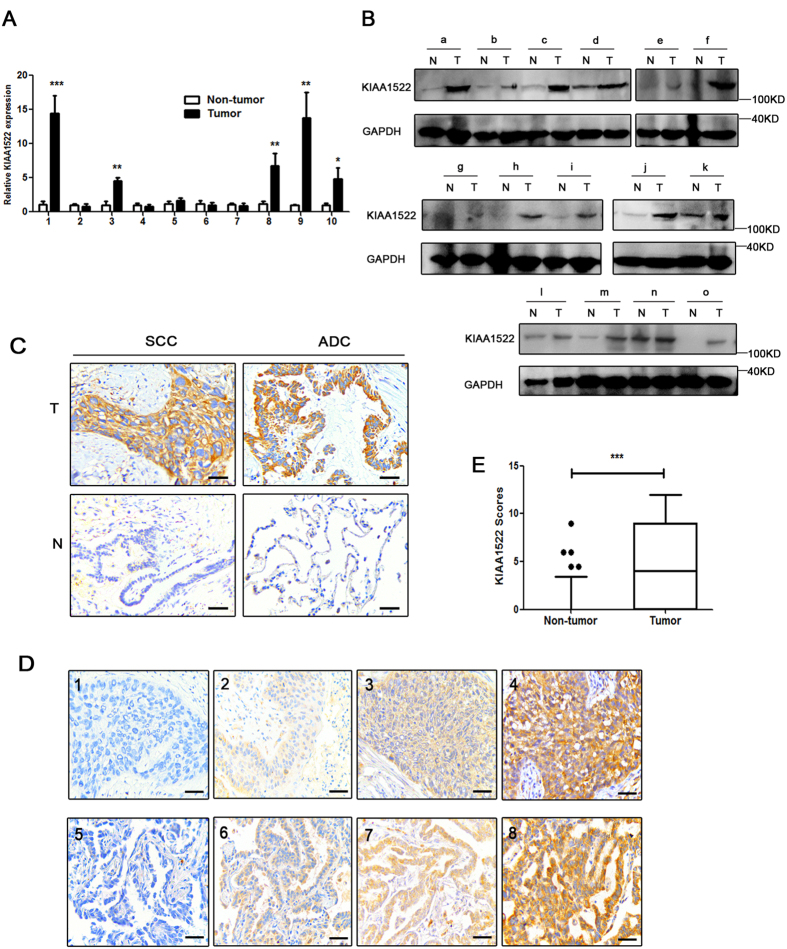
Expression status of KIAA1522 protein in NSCLC tissue samples. (**A**) The mRNA levels of KIAA1522 in 10 pairs of NSCLC tissue samples relative to their adjacent non-malignant tissues measured by real-time PCR. The expression of GAPDH mRNA was used as loading control in the real-time PCR analysis. (**B**) Western blotting analysis of KIAA1522 protein levels in NSCLC tissue samples (**T**) and the matched non-tumor tissues (**N**). GAPDH was used as loading control in the immunoblotting assays. (**C**) Representative examples of high (positive) and low (negative) expression of KIAA1522 in SCC or ADC samples and their adjacent non-malignant tissues. (**D**) Representative immunohistochemical microphotographs of KIAA1522 with negative (1,5), weak (2,6), moderate (3,7) and strong (4,8) expression. The subtypes are SCCs (1–4) and ADCs (5–8). Bar = 100 μm. (**E**) The expression scores indicating the protein levels of KIAA1522 for NSCLC and non-tumor lung tissues. Boxes represent the upper and lower quartiles and median; whiskers show the data points that are neither lower than the first percentile nor greater than the 99th percentile (by Mann-Whitney test analysis, ***P < 0.001).

Real-time PCR and immunoblotting assays indicated the elevated KIAA1522 mRNA and protein levels in part of NSCLC tissue samples compared to the adjacent non-tumor tissues (operative margins) (Fig. 2A,B). To test the protein level of KIAA1522 in a relatively large sample size and to examine the potential relevance with clinical parameters, immunohistochemistry (IHC) assays were performed to detect KIAA1522 expression in tissue microarrays (TMAs) containing 583 NSCLC tissues and their paired non-tumor tissues. KIAA1522 protein expression in tumor tissues was observed in both cytoplasm and cell membrane. The protein displayed strong expression in 156 NSCLC (71 SCCs and 85 ADCs) cases, but showed no or weak staining in adjacent non-neoplastic tissues. Representative images of expression of KIAA1522 in SCC or ADC samples are exhibited in Fig. 2C. And the indicative examples of each level of staining were shown in Fig. 2D. Statistical analysis revealed that KIAA1522 protein was significantly overexpressed in NSCLC tissues than in non-tumor tissues (Fig. 2E). Overexpression of KIAA1522 was found more in female than male patients (31.3% vs. 26.1%; *P* = 0.228), and more in ADCs than SCCs (31.1% vs. 24.1%; *P* = 0.060). KIAA1522 was detected a little more in current or former smokers than in nonsmokers (27.5% vs. 27.3%; *P* = 0.963). Late-stage (stage III-IV) patients showed more positive expression of KIAA1522 than early-stage ones (32.1% vs. 25.5%; *P* = 0.106).

### KIAA1522 mRNA correlates with overall survival (OS) of NSCLC patients

**Figure 3:**
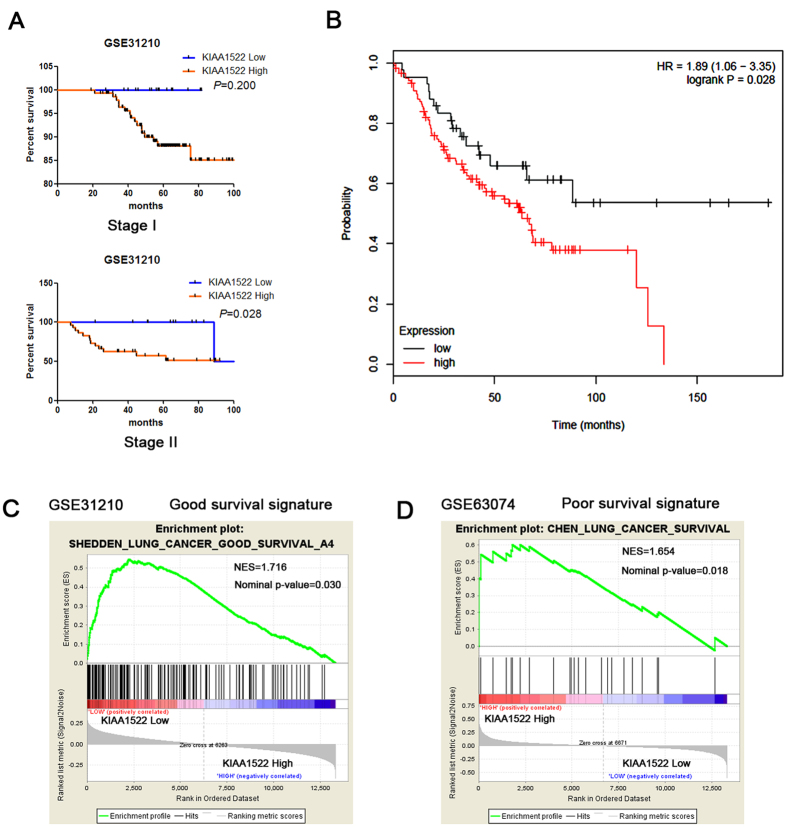
Correlation of KIAA1522 mRNA in NSCLC tissues and patients’ survival/survival signatures. (**A**) Kaplan-Meier curves showing the survival percentage of patients with high KIAA1522 mRNA and low KIAA1522 mRNA levels in stage I and II patients in the dataset GSE31210. Samples with incomplete resection or adjuvant therapy were excluded for prognosis analysis. *P* values are shown in the graph, by log-rank test. (**B**) Survival analysis of the stage II lung cancer patients with KIAA1522 high and low expression using the Kaplan-Meier Plotter website for lung cancer (Version 2015). (**C**,**D**) Gene set enrichment analysis in GSE31210 showed that the genes within a good survival signature were observed to enrich in the groups with low KIAA1522 expression (**C**), while analysis in GSE63074 illustrated the enrichment of poor survival signature genes in the subset of high KIAA1522 expression group (**D**).

To delineate the correlation of KIAA1522 mRNA in NSCLC tissues with OS in NSCLC patients, a publicly available transcriptome dataset (GSE31210) was analyzed. From the total dataset of 226 stage I and stage II samples, 22 patients with incomplete resection or adjuvant therapy were excluded, leaving 204 patients for analysis. The survival analysis revealed that in stage II lung cancer patients, the ones with KIAA1522 high expression represented shorter overall survival than those with low KIAA1522 expression (*P* = 0.028, Fig. 3A). Survival analysis was also performed through a website based software, the Kaplan-Meier Plotter^16^, the survival curves derived from a collection of the stage II patients from multi-datasets (all datasets provided by the website) revealed the similar results (Fig. 3B). To further corroborate the prognostic role of KIAA1522, we performed the gene set enrichment analysis (GSEA) using NSCLC datasets with previously annotated gene signatures associated with good or poor survival. We found the enrichment of the genes in a good survival signature was observed in the groups with low KIAA1522 expression (Fig. 3C), while the enrichment of a group of poor survival signature genes in the subset of high KIAA1522 expression group (Fig. 3D).

### KIAA1522 protein expression and overall survival (OS) of patients

Besides transcript levels, the protein levels of KIAA1522 detected by IHC were also examined for the prognostic roles in NSCLC patients. In tissue samples, Kaplan-Meier analysis indicated that patients with high expression of KIAA1522 had a lower OS compared to those with low expression of the protein, the difference was significant for the patients at all stages (*P* < 0.00001) as well as for the ones at early-stages (*P* = 0.00011) and late-stages (*P* = 0.039).

**Figure 4:**
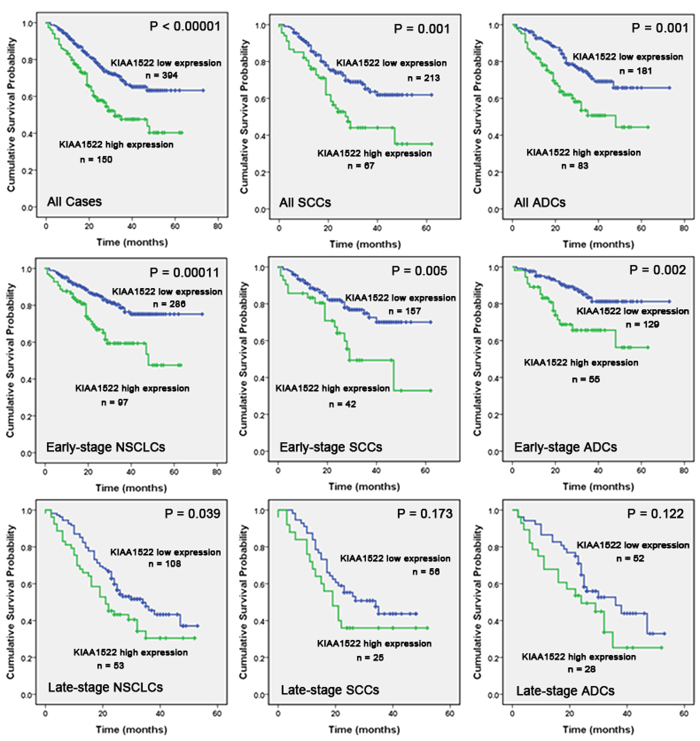
Relationship between expression of KIAA1522 and patient OS. Kaplan-Meier curves showing the association between expression of KIAA1522 and patients OS in all stages, in the stratified stages and in different histological tumor types in tissue samples (all of the *P* values are shown in the graph, by log-rank test).

When considering the different histological types of tumor, the OS of the KIAA1522 overexpression group was shorter than that of the KIAA1522 low expression group in both SCCs and ADCs for all stages (*P* = 0.001, 0.001) and early stages (*P* = 0.005, 0.002) (Fig. 4).

### Effect of KIAA1522 expression on survival by Cox regression analysis

Univariate Cox regression analyses of the prognostic significance showed that overexpression of KIAA1522 was significantly associated with an elevated risk of death compared to low expression of the protein (*P* = 0.00001, HR = 2.003, 95%CI: 1.475–2.719). Male (*P* = 0.023), late-stage patients (*P* < 0.00001), tumor size >7cm (*P* < 0.00001), Lymph node metastasis (*P* < 0.00001) and poorly differentiated tumors (*P* = 0.025) were also contribute factors to shorter OS of patients. Multivariate Cox proportional hazards model indicated that KIAA1522 was an independent prognostic factor in tumor tissues as compared with sex, stage, tumor size, N-status and tumor differentiation (*P* = 0.00003, HR = 1.942, 95%CI: 1.425–2.647, Table 1).

Furthermore, we evaluated whether KIAA1522 expression showed independent prognostic significance in 410 early-stage (stages 0-II) NSCLCs. Multivariate Cox regression analysis indicated that KIAA1522 was also an independent prognosticator in these early-stage NSCLCs (*P* = 0.00025, HR = 2.317, 95%CI: 1.477–3.635; Table 2).

### KIAA1522 predicts responsiveness of platinum-based chemotherapy

**Figure 5:**
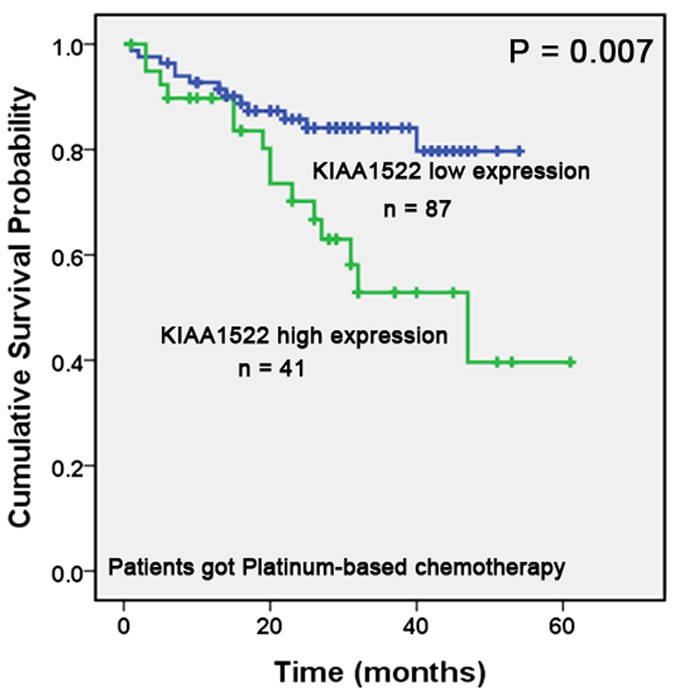
Relationship between expression of KIAA1522 and overall survival of patients with platinum-based chemotherapy. Kaplan-Meier curves showing that patients with high expression of KIAA1522 had a poorer OS compared to those with low expression of the protein (*P* = 0.007, by log-rank test).

In our study, 131 patients (Median 58, range 39–75) got platinum-based chemotherapy in later clinical course after tumor resection in the Cancer Hospital, CAMS. There were 89 males and 42 females with 60 SCCs and 71 ADCs. And 91 patients were diagnosed as early stage and 40 were at late-stage. We analyzed the association between KIAA1522 expression and overall survival of the patients in order to evaluate whether KIAA1522 expression could identify patients who benefit from chemotherapy. We found that patients with low expression of KIAA1522 had a longer OS compared to those with high expression of the protein, the difference was significant (*P* = 0.007) (Fig. 5).

### Correlation of KIAA1522 expression with KRAS pathway activation

**Figure 6:**
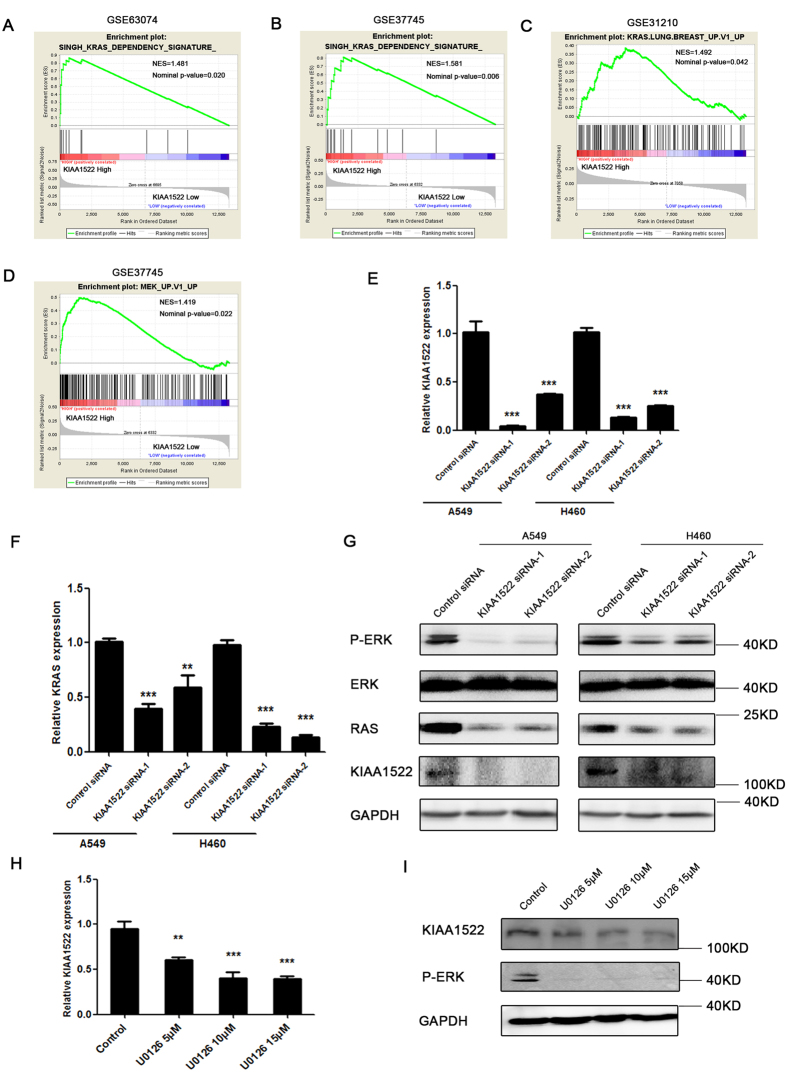
Correlation of KIAA1522 expression with KRAS pathway signatures. (**A**–**C**) Gene sets enrichment analysis of the NSCLC dataset showing that high KIAA1522 expression was associated with the hyper-activation of KRAS signatures in dataset GSE63074 (**A**), GSE37745 (**B**) and GSE 31210 (**C**). (**D**) KIAA1522 high expression showed positive correlation with activation of MEK signaling in GSE37745. (**E**) The expression of KIAA1522 in the A549 and H460 cells that were transfected with control siRNA or two independent KIAA1522 siRNAs for three days. Data are shown as mean ± s.d. (by *t-*test analysis, *** *P* < 0.001, n = 3). (**F**) The relative KRAS mRNA levels in the A549 and H460 cells transfected with control siRNA or two independent KIAA1522 siRNAs for three days were analyzed by real-time PCR. Data shown are mean ± s.d. (by *t-*test analysis, ***P < 0.001, **P < 0.01, n = 3). (**G**) Western blotting analysis of the Phospho-ERK (Thr202/Tyr204), ERK, RAS, KIAA1522 and GAPDH (loading control) levels in the A549 and H460 cells transfected with the indicated siRNAs, four days after transfection. (**H**–**I**) The H460 cells were treated with DMSO (control) or the indicated concentration of U0126 for 24 hours; the cells were then subjected for real-time PCR (H) and western blotting (I) analysis of KIAA1522 expression. The efficiency of MEK inhibitor U0126 were revealed by the reduced Phospho-ERK (Thr202/Tyr204) levels tested by western blot. Data shown are mean ± s.d. (by *t-*test analysis, **P < 0.01, ***P < 0.001, n = 3).

**Figure 7:**
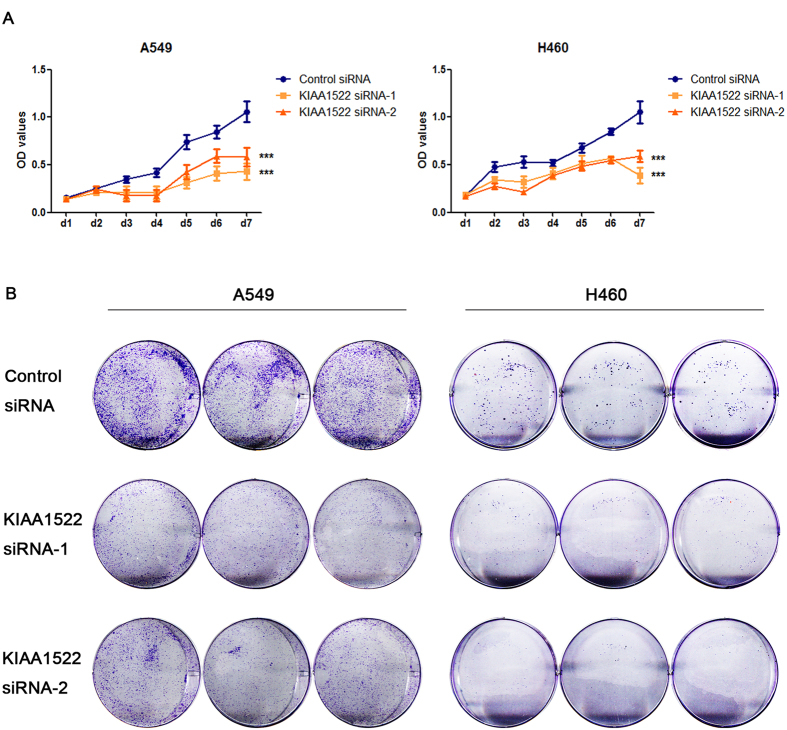
Knockdown of KIAA1522 expression inhibits the growth of NSCLC cells. (**A**,**B**) The growth rate of NSCLC cell lines A549 and H460 transfected with the indicated siRNAs were tested by the MTT assays (**A**) and colony formation assays (**B**). Data shown are mean ± s.d. (by ANOVA analysis, ***P < 0.001, n = 3).

To get further insight into the biological roles of KIAA1522, GSEA was used to examine whether the KIAA1522 gene was involved in some oncogenic pathways. After screening the association of KIAA1522 expression with the oncogenic signatures within the “c6.all.v5.0.” gene sets from MSigDB database^17^, the results revealed that high KIAA1522 expression was associated with the hyper-activation of KRAS signatures in three datasets (Fig. 6A–C) and the activation of MEK signaling which is a down-stream factor of KRAS (Fig. 6D), suggesting the involvement of KIAA1522 in KRAS pathways. To test the association of KIAA1522 expression with the activation of KRAS pathway, the loss of function assays were performed in lung cancer cell lines A549 and H460, both of which express the mutant form of KRAS gene, i.e. the oncogenic KRAS. The results showed that knockdown of KIAA1522 expression (Fig. 6E) in A549 and H460 cell lines down-regulated the mRNA levels of the oncogenic KRAS (Fig. 6F). Also, western bolt assay showed that inhibition of KIAA1522 expression could reduce RAS protein and resulted in the lower levels of phosphor-ERK protein, which indicating the inhibition of oncogenic RAS signaling (Fig. 6G). On the other hand, the inhibition of RAS-MEK-ERK signaling through the treatment of the cells with MEK specific inhibitor U0126 resulted in the reduced KIAA1522 expression (Fig. 6H,I). Furthermore, the loss of KIAA1522 function suppressed the growth of lung cancer cell lines, which was revealed by both the MTT and colony formation assays (Fig. 7A,B). Taken together, the positive regulation of cell growth and oncogenic RAS signaling pathway by KIAA1522 were in line with the potential oncogenic functions of this poor survival indicator.

## Discussion

The assessment of prognosis for cancer patients is crucial in clinical course to the selection of high-risk patients who would benefit from neoadjuvant chemotherapy or other appropriate treatment. The widely accepted ways to find biomarkers for a certain cancer type are mainly attributed to two categories: One is to examine the aberrant expression and/or the prognostic value of a specific gene or a panel of genes with important biological functions^18^. This methodology is certainly reasonable, but this approach may prevent some uncharacterized genes with prognostic and diagnostic values from uncovering. Another way is based on some high-throughput technology to search biomarkers at the genomic or the proteomic scales^10^,^19^,^20^. These methods often cost a lot and may not be easily applied to clinical practice. Take the advantage of the availability of online expression profile datasets, data mining approach was also utilized to search potential biomarkers^11^,^21^. Likewise, we screened a series of independent microarray datasets on the focus of genes with little functional annotations and identified the gene of KIAA1522 as an aberrantly overexpressed gene in lung cancer tissue samples. The following IHC experiments and statistical studies indicated that in consistent with mRNA, the up-regulation also occurred in KIAA1522 protein levels. And the prognostic role attributed to both the mRNA and protein of this gene (Figs 3 and 4). These findings implicated that our approach was successful in discovering novel biomarkers.

The value of KIAA1522 as a potential biomarker for clinical usage relied on its specificity of immuno-staining and easy to detect in clinical practice. The high specificity of this protein in immune-staining was reinforced by our previous report that KIAA1522 protein consisted in a six-protein panel detected in bronchial brushings could act as diagnostic marker for early detection of lung cancer^15^. In our present study, KIAA1522 is highly specific to lung cancer tissues but with little/no positive signals detected in non-tumor lung tissue samples. Moreover, the staining signals of KIAA1522 protein is clearly observed in cytoplasm and cell membrane of lung cancer tissue species, which make it clinical amenable to differentiate between different levels of staining (Fig. 2D). Also, its detecting technology-immunohistochemistry, is a convenient test without requiring some expensive facilities, and is widely used in clinical practice and available in most laboratories.

Nowadays, even the patients of NSCLC was detected at early stage, the curable patients by solely surgical resection remains unsatisfactory^19^,^22^,^23^. It is worthy to note that KIAA1522 could be used as an independent prognostic marker even in early-stage NSCLC patients, which may provide useful information for doctors to make optimal clinical decisions and assigned those patients with potential poor prognosis to more appropriate treatment.

Adjuvant platinum-based chemotherapy remains the mainstay of treatment for non-small cell lung cancer. Though many predictive markers have been assessed^24^,^25^,^26^,^27^,^28^,^29^, no molecular marker has been shown to be useful for patient selection until recently. Besides the prognostic role, our present work also showed that high expression of KIAA1522 predicted poor responses for platinum-based chemotherapy, making it a potential biomarker of platinum-resistance. The KRAS pathway (no matter mutation or not) is a well known oncogenic signaling in lung cancer, which contributes to multiple aspects of malignancy including drug resistance^30^,^31^,^32^. The activation of KRAS through mutation has been reported to be predictive of poor survival in lung cancer patients^33^,^34^, and also correlated with chemotherapy sensitivity^35^. In agreement with the prognostic role of KIAA1522 and its association with chemo-sensitivity, we found the enrichment of KRAS down-stream signaling genes within KIAA1522 high expression groups of lung cancer and the reciprocal regulation between KIAA1522 expression level and the activation of oncogenic RAS signaling *in vitro* (Fig. 6). These results not only made our conclusion more convincing but also implicated the involvement of this gene in the oncogenic KRAS signaling in lung cancer cells. However, the precise role of this gene in the regulation of KRAS pathways requires further studies. Besides, several established or developing targeting therapy strategies^36^,^37^ are based on the inhibition of KRAS signaling, in view that KIAA1522 associated with KRAS pathways, this biomarker is more likely to predict responses to those treatments, for achieving that, additional independent and prospective validation studies are needed.

## Methods

### Ethics statement

This study was approved by the Ethics Committee/Institutional Review Board of the Cancer Institute (Hospital), PUMC/CAMS (No. 12-098/632). Written informed consent forms were obtained from patients for sampling and research. And all the methods in our study were carried out in accordance with the approved guidelines.

### Patients and samples

All tissue samples were procured in the Cancer Hospital, Chinese Academy of Medical Sciences and Peking Union Medical College (CAMS & PUMC) from 2005 to 2010, which were 583 surgically resected NSCLC tissues (303 SCCs and 280 ADCs) and matched morphologically normal operative margin. All samples in this study were from Asian patients. Of all the tissue samples in our study, 410 (70.3%) were at early stage (stages 0-II) with 215 SCCs and 195 ADCs. The tissue samples were collected shortly after radical surgery of NSCLC patients. Primary tumor regions and adjacent non-neoplastic tissues were excised and pathological diagnoses were made by experienced pathologists. For construction of microarrays (TMA), tissues were routinely fixed with neutral buffered formalin (pH 7.4) and paraffin-embedded. All of the samples in this study were residual specimens after diagnostic sampling. None of the patients received treatment prior to surgery. 131 patients received further adjuvant Platinum-based chemotherapy after surgery in the Cancer Hospital, CAMS. Basic features of the patients are recorded concerning the clinical/pathological parameters of tumors (Table 3). Survival data were available with a median follow-up of 754 days (range 21~2,190 days).

### Cell culture, transfection and reagents

The human lung cancer cell lines A549 and NCI-H460 were acquired from the American Type Culture Collection (ATCC, Manassas VA, USA). Cell lines were maintained at 37 °C in 5% CO_2_ in Dulbecco’s modified Eagle medium supplemented with 10% fetal bovine serum. Transfection was performed using the Lipofectamine® 2000 Transfection Reagent from Invitrogen. The MEK inhibitor U0126 was acquired from Cell Signaling Technology. Inc. The duplex siRNAs were synthesized by Genepharma Company (Shanghai, China). The according DNA sequences of siRNAs which were used to specifically knock down KIAA1522 expression were 5′-GGCTGAGAATGACAAACAT-3′ and 5′- CATGACTCATTTCCCAAAT-3′.

### The expression profile datasets

Gene expression datasets used for statistical analysis were acquired from the National Center for Biotechnology Information gene expression omnibus database with the accession codes GSE19804^38^, GSE32863^39^, GSE19188^40^, GSE31210^41^,^42^, GSE37745^43^ (analysis were performed in the no recurrence subgroup of GSE37745) and GSE63074.

### Screen of uncharacterized genes that overexpressed in NSCLC datasets

The screening was performed in GSE19804 and GSE32863 which consist of lung tumor samples and non-tumor lung samples. In both datasets, the probes were chosen for screening following two criteria: 1. the probe specified an uncharacterized gene without GO annotation; 2. the average of normalized expression value (log2 transformed value) of the probe in tumor samples is more than 9.5. The average value of log_2_ (Tumor/Non-tumor) was calculated for each selected probe and listed in the rank order. The identified overexpressed genes had positive log_2_ (Tumor/Non-tumor) value in both datasets while selected.

### Real-time PCR

Total RNA from tissues and cells were extracted through the RNAiso Plus kit (Takara Bio Inc.) and the complementary DNA was generated using the primeScript RT Master kit (Takara Bio Inc.). The reverse transcription was performed under 37 °C for 1 hour. Real-time PCR was performed by SYBR Green PCR Master Mix (AB Applied Biosystems). The conditions of PCR were as follows: 50 °C for 2 minutes, then 95 °C for 2 minutes, followed by 40 cycles of amplifications, including 95 °C for 15 seconds and 60 °C for 1 minute. The primers used in quantitative detection of gene expression were as follows: human GAPDH-F: CATGAGAAGTATGACAACAGCCT, human GAPDH-R: AGTCCTTCCACGATACCAAAGT; human KIAA1522-F: CAAGAGGGCCAAGGGCAAAG, human KIAA1522-R: GGTCGCCCACTGGGAAAGAA; human KRAS-F: TCCCAGGTGCGGGAGAG, human KRAS-R: TTAGCTGTATCGTCAAGGCACT. There is no non-specific amplification determined by dissolved curves.

### Western blot assay

Cell lines or tissues were lysed with RIPA (Thermo Fisher Scientific, 89901) containing protease inhibitor cocktail (Roche Diagnostics, 05892970001) and phosphatase inhibitor cocktail (Roche Diagnostics, 04906845001). The total protein concentration was estimated using a BCA protein assay kit (Thermo Fisher Scientific, 23225). Proteins were then separated by SDS-PAGE followed by transferred to NC membranes (Pall Corporation) and detected by the primary and secondary antibodies.

The primary antibodies used in the western blot assays were Phospho-p44/42 MAPK (Erk1/2) (CST, #4370, 1:1000), p44/42 MAPK (Erk1/2) (CST, #4695, 1:1000), RAS (Millipore, 05–516, 1:1000), KIAA1522 (Sigma, HPA032050, 1:500; Biosynthesis Biotechnology, bs-8563R, 1:300) and GAPDH (Santa Cruz, sc-25778, 1:1000). The secondary antibodies used in the western blotting assay were goat anti-rabbit (Santa Cruz, sc-2004) or goat anti-mouse (Santa Cruz, sc-2005) HRP (horseradish peroxidase)-conjugated secondary antibodies. The SuperSignal West Dura Extended Duration Substrate (Thermo Fisher Scientific, 34076) was used to visualize the blots.

### Tissue microarrays (TMA) construction and immunohistochemistry (IHC)

The TMA was constructed as described previously^44^. For each case, three cancer tissue cores (diameter = 1 mm; height = 5 mm) and two matched adjacent non-neoplastic tissue cores were taken from the primary block.

IHC was performed on the 4-μm sections of the resulting TMA block. The slides were deparaffinized, rehydrated, immersed in 3% hydrogen peroxide solution for 15 min, heated in citrate buffer (pH 6.0) for 25 min at 95 °C, and cooled for 60 min at room temperature. Between each incubation step, three times of washings with PBS (pH 7.4) were carried out. After blocked with 10% normal goat serum for 30 min at 37 °C and washed, the slides were incubated overnight at 4 °C with rabbit polyclonal antibody against KIAA1522 (1:200; HPA032050, Sigma ImmunoChemicals, St Louis, MO, USA) and visualized using the PV-9000 Polymer Detection System following the manufacturer’s instructions (GBI, USA). After washing with PBS, the slides were counterstained with hematoxylin.

### Immunohistochemical assessment

The results of immunohistochemical and staining were scored blindly with no information of the clinical data. Protein expression levels were determined on the basis of staining intensity and the percentage of immunoreactive cells. Staining intensity was rated as 0 (negative), 1 (weakly positive), 2 (moderately positive), and 3 (strongly positive). The percentage of immunoreactive cells was graded as 0 (0%), 0.5 (1–10%), 1 (11–20%), 2 (21–50%), 3 (51–80%), or 4 (81–100%). The average of tumor cell staining intensity score multiplied by the percentage of positive cells score represented the final score of the specimens. All cases were divided into two groups, a strongly positive group (score range, 9–12) and a low/no expression group (score range 0–9).

Assessment and imaging of IHC was performed using a Leica DM2000 microscope equipped with Leica DFC Cameras-Image Acquisition System (software V3.5.0, Switzerland).

### Gene Set Enrichment Analysis

Gene Set Enrichment Analysis (GSEA) was performed using the GSEA program provided by the Broad Institute (http://www.broadinstitute.org/gsea/index.jsp). GSEA compared the expression levels of the genes within each indicated geneset between KIAA1522 high expression and low expression groups and to examine the relative enrichment of the genes in a specific group. The genesets used for analysis were downloaded from the Molecular Signatures Database^17^.

### MTT assay

Cells were seeded at 1000 cells in 200 μL DMEM per well in 96-well culture plates. At the indicated time points, 20 μL of 0.5 mg/ml MTT (Thiazolyl Blue Tetrazolium Bromide, M5655, Sigma) was added to each well. After the incubation at 37 °C for three hours, the culture in each well was replaced with 150 μL DMSO (dimethyl sulphoxide, D8418, Sigma). The absorbance values (OD 590 nm) were measured using a spectrophotometer (Thermo Fisher Scientific). The growth curves were shown to reveal the growth rates.

### Colony formation assay

Cells were seeded in 2 ml DMEM per well in the 6-well culture plates (5000 cells per well for A549 and 2000 cells per well for H460). After 10 days’ culture, cells were fixed with methanol and stained with crystal violet (Beyotime, C0121).

### Statistical analysis

The analyses were performed using PASW Statistics 18 (SPSS Inc., Chicago) or GraphPad Prism 5 software (GraphPad Software, Inc., La Jolla, CA). Associations between protein expression and clinicopathologic parameters were assessed by the Mann–Whitney test and the Kruskal–Wallis test. Difference between the gene expression levels within tumor and non-tumor tissues or different groups of cells were analyzed using students *t*-test or the Mann–Whitney test. Results from MTT assays were analyzed using ANOVA analysis. For survival analyses, Kaplan-Meier survival curves were constructed, and differences were tested by the log-rank test. Overall survival was defined as the time between the date of surgery/the date of receiving first cycle of platinum-based chemotherapy and the date of death from lung cancer or the date of last contact. Survival analysis was also performed using the Kaplan-Meier Plotter website for lung cancer (Version 2015) (http://kmplot.com/analysis/index.php?p=service&cancer=lung)^16^. Univariate and Multivariate Cox proportional hazards regression models were performed to identify the independent factors with a significant impact on patient survival. The hazard ratios (HRs) and 95% confidence intervals of the prognostic factors were calculated. All *P* values were two-sided, and the results were considered significant if *P* < 0.05.

## Additional Information

**How to cite this article**: Liu, Y.-Z. *et al.* KIAA1522 is a novel prognostic biomarker in patients with non-small cell lung cancer. *Sci. Rep.* **6**, 24786; doi: 10.1038/srep24786 (2016).

## Figures and Tables

**Table 1:** Univariate and multivariate analysis of survival in all NSCLC patients.

Variable	Univariate Analysis			Multivariate Analysis		
	HR	95%CI	*P*	HR	95%CI	*P*
KIAA1522						
High vs. low expression	2.003	1.475–2.719	0.00001	1.942	1.425–2.647	0.00003
Age						
≥60 vs. <60	1.327	0.983–1.792	0.065			
Sex						
Male vs. female	1.524	1.061–2.188	0.023	1.782	1.229–2.583	0.002
Tumor type						
SCC vs. ADC	1.293	0.963–1.737	0.088			
Stage						
0+I+II vs. III+IV	2.643	1.971–3.544	<0.00001	1.679	1.070–2.633	0.024
T status						
≤7 cm vs. >7 cm	2.147	1.586–2.907	<0.00001	1.550	1.082–2.220	0.017
N status						
N0 vs. N1-3	2.199	1.621–2.984	<0.00001	1.577	1.043–2.386	0.031
M status						
M0 vs. M1	1.405	0.577–3.421	0.455			
Tumor differentiation						
Well vs. Moderate vs. poorly	1.339	1.037–1.728	0.025	1.105	0.845–1.446	0.466
Gross pathology						
Central vs. peripheral	1.232	0.915–1.658	0.169			
Smoking history						
Non vs. current or former smoker	1.230	0.887–1.707	0.215			

HR = Hazard Ratio.

**Table 2:** Univariate and multivariate analysis of survival in early-stage NSCLC patients.

Variable	Univariate Analysis			Multivariate Analysis		
	HR	95%CI	*P*	HR	95%CI	*P*
KIAA1522						
High vs. low expression	2.304	1.489–3.567	0.00018	2.317	1.477–3.635	0.00025
Age						
≥60 vs. <60	2.006	1.254–3.207	0.004	2.082	1.269–3.415	0.004
Sex						
Male vs. female	2.459	1.307–4.626	0.005	2.188	1.042–4.595	0.039
Tumor type						
SCC vs. ADC	1.601	1.044–2.457	0.031	1.319	0.815–2.135	0.260
T status						
≤7 cm vs. >7 cm	1.707	0.964–3.022	0.067			
N status						
N0 vs. N1-3	1.528	0.983–2.377	0.060			
Tumor differentiation						
Well vs. Moderate vs. poorly	1.128	0.801–1.589	0.490			
Gross pathology						
Central vs. peripheral	1.511	0.989–2.308	0.056			
Smoking history						
Non vs. current or former smoker	2.010	1.167–3.461	0.012	1.006	0.518–1.952	0.986

HR = Hazard Ratio.

**Table 3:** Basic clinicopathologic data of tissue samples from patients with NSCLC.

Parameter	No. of tissue samples (%)
Age-yr	
Median	61
Range	31–84
Sex	
Male	434 (74.4)
Female	149 (25.6)
Tumor type	
SCC	303 (52.0)
ADC	280 (48.0)
Tumor stage^a^	
0	1 (0.2)
I	187 (32.1)
II	222 (38.0)
III+IV	173 (29.7)
T status	
Tis	1 (0.2)
T1	73 (12.5)
T2	373 (64.0)
T3	96 (16.4)
T4	40 (6.9)
N status	
N0	304 (52.1)
N1-3	279 (47.9)
M status	
M0	572 (98.1)
M1	11 (1.9)
Tumor differentiation^b^	
Well	30 (5.2)
Moderate	270 (46.3)
Poorly	283 (48.5)
Gross pathology	
Central-type	314 (53.9)
Peripheral-type	269 (46.1)
Smoking status	
Nonsmoker	179 (30.7)
Current or former smoker	398 (68.3)
Unknown	6 (1.0)

^a^Tumor stage was classified according to the 7th edition of the International Union against Cancer (UICC) Tumor Node Metastasis (TNM) classification of malignant tumors.

^b^Tumor differentiation was based on the criteria of the 2004 World Health Organization Classification of Tumors.
